# Associations between vitamin D status and sight threatening and non-sight threatening diabetic retinopathy: a systematic review and meta-analysis

**DOI:** 10.1007/s40200-022-01059-3

**Published:** 2022-05-26

**Authors:** Mike Trott, Robin Driscoll, Enrico Iraldo, Shahina Pardhan

**Affiliations:** 1 Vision and Eye Research Institute (VERI), Anglia Ruskin University, Young Street, Cambridge, CB1 2LZ UK; 2 Centre for Public Health, Queen’s University Belfast, Belfast, UK

**Keywords:** Vitamin D, Dietetics, Nutrition, Diabetes, Retinopathy

## Abstract

**Background:**

Vitamin D levels have been shown to be associated with diabetic retinopathy, however to date, no review has examined the relationship between vitamin D and sight threatening diabetic retinopathy (STDR) and non-sight threatening diabetic retinopathy (NSTDR). The aim of this review, therefore, was to pool associations between vitamin D deficiency (25(OH)D < 20 ng/mL) and STDR/NSTDR. A further aim was to examine associations between circulating 25(OH)D levels and STDR/NSTDR.

**Methods:**

A systematic review of major databases was undertaken for studies published from inception to 22/04/2022, using a pre-published protocol. Studies reporting prevalence of STDR or NSTDR versus a control group with diabetes and no DR or DME and either (a) vitamin D deficiency prevalence, or (b) circulating 25(OH)D levels, were included. A random effects meta-analysis was undertaken.

**Results:**

Following screening, 12 studies (*n* = 9057) were included in the meta-analysis. STDR was significantly associated with vitamin D deficiency (OR = 1.80 95%CI 1.40–2.30; *p* = <0.001), whereas NSTDR was not (OR = 1.07 95%CI 0.90–1.27; *p* = 0.48). Both conclusions were graded as low credibility of evidence. Furthermore, circulating 25(OH)D levels were significantly associated with both NSTDR (SMD = -0.27 95%CI -0.50; −0.04; *p* = 0.02) and STDR (SMD = −0.49 95%CI -0.90; −0.07; *p* = 0.02), although these were graded as low credibility of evidence.

**Conclusion:**

Vitamin D deficiency is significantly associated with STDR (including DME), but not with NSTDR. Given the well-reported associations between vitamin D deficiency and other unfavourable outcomes, it is important that vitamin D deficiency is managed appropriately and in a timely manner to reduce the risk of blindness in people with diabetes.

**Supplementary Information:**

The online version contains supplementary material available at 10.1007/s40200-022-01059-3.

## Introduction

Vitamin D is a major contributor to the regulation of calcium and phosphate in the body, and has been associated with several conditions, including autoimmune disorders, immune function, and inflammation [1–3]. Vitamin D deficiency can be defined as having plasma 25-hydroxyvitamin D (25(OH)D) levels of below 20 ng/mL (equivalent to <50 nmol/L) [1]. Moreover, it has been reported that insufficient concentrations of vitamin D is a significant risk factor for mortality [4, 5]. Vitamin D, as well as several other dietary components [6, 7], has been associated with several types of diabetic outcomes/complications; indeed, people with diabetes mellitus have been shown to have higher levels of inflammatory markers [8, 9], especially in people with associated diabetic microvascular complications [10–12], including diabetic retinopathy (DR).

DR can be characterised by changes in the eye causing visual impairment, and eventually blindness if left untreated [13]. DR is one of the leading cause of blindness among people with diabetes [14]. DR is commonly classified under the Early Treatment of Diabetic Retinopathy Study (ETDRS) criteria [15], which grades DR according to severity. In brief, the scale classifies DR into discrete categories including no DR, mild non-proliferative DR (NPDR), moderate NPDR, severe NPDR, proliferative DR (PDR) [16]. Furthermore, the presence of diabetic macular edema (DME) can affect the eyesight regardless of DR status [16]. It has been reported that DR can be categorised as sight threatening (STDR) and non-sight threatening (NSTDR), with several studies using the following criteria for STDR: the presence of severe NPDR, pre-PDR, PDR, or the presence of DME, regardless of DR status [17–19]. The criteria for NSTDR has been the presence of mild and moderate NPDR [17].

Several systematic reviews with meta-analyses have reported on the association between vitamin D status and DR, with one review reporting significant associations between DR and vitamin D deficiency (odds ratio (OR) = 1.35), PDR and vitamin D deficiency (OR = 1.69), and between DR and 25(OH)D levels (pooled mean difference = −1.68) [20]. A key limitation to this review is that several of the included studies had different criteria for vitamin D deficiency (for example vitamin D deficiency cut-offs ranged from <20 mg/mL to <30 ng/mL), making the included studies highly heterogeneous. Another systematic review found similar significant associations between vitamin D deficiency and NPDR (OR = 1.21) and PDR (OR = 1.32) [21], however both of these reviews did not stratify between STDR and NSTDR. Furthermore, neither study included DME in their analyses. It was therefore the primary aim of this review to examine associations between vitamin D deficiency and STDR/NSTDR. A secondary aim of this review is to examine associations between 25(OH)D levels and STDR/NSTDR. This review has the potential to provide more information on the links between vitamin D deficiency and DR using strict criteria, and can inform future research and inform medical recommendations and policy.

### Methods

This systematic review was conducted in accordance with the Preferred Reporting Items for Systematic Reviews and Meta-Analyses (PRISMA) guidelines [22], and has been registered with the international prospective register of systematic reviews (PROSPERO protocol ID CRD42021257772). There were no deviations from the published protocol.

### Search strategy

Databases were searched from inception to 22/04/2022 including Pubmed; Embase; OpenGrey; CINAHL; the Cochrane Library; and Web of Science, using the following search terms:

(vitamin D OR cholecalciferol OR 25-hydroxyvitamin D OR 25(OH)D)

AND

(diabetic retinopathy OR diabetic macular edema OR diabetic macular oedema OR proliferative diabetic retinopathy OR proliferative retinopathy OR sight threatening retinopathy OR retinopathy)

No other limiters were applied.

Results of searches were imported in a bibliographic database, with duplicates removed automatically. Titles and abstracts of the remaining studies were independently screened for inclusion by two authors (MT; EI). Following title and abstract screening, the full texts of all potentially eligible papers were reviewed independently by two reviewers (MT; EI) before making a final decision on eligibility, with a senior reviewer (SP) mediating any disputes. The following section describes the inclusion and exclusion criteria:

#### Population

People of any age with diabetes (Type 1 or Type 2) were considered.

#### Intervention(s)/exposure(s)

Studies were required to report the prevalence of STDR or NSTDR versus a control group with diabetes and no evidence of DR. In line with previous studies, STDR was defined as the presence of any of the following: severe non-proliferative, pre-proliferative, or proliferative retinopathy. The presence of diabetic macular edema (DME) was also classified as STDR regardless of DR status. NSTDR was defined at the presence of mild or moderate non-proliferative DR [17].

#### Comparator(s)/control(s)

Studies were required to include either:

1. Data regarding vitamin D deficiency prevalence (defined as 25(OH)D levels of <20 ng/mL or < 50 nmol/L), or
2. Circulating levels of 25(OH)D as a continuous variable

#### Outcomes

Studies had to report one or more of the following:

1. Odds ratio (OR) of STDR/NSTDR risk versus no DR (or yielded data so that an OR could be calculated) in groups with versus without vitamin D deficiency
2. Mean 25(OH)D levels of STDR/NSTDR versus no DR

Furthermore, studies were also excluded if they:

1. Were written in languages other than English, Italian, French, or Spanish
2. Had not been through the peer-review process (for example, pre-prints)

### Data extraction

Data were extracted by two reviewers (MT; RD) and included: first author; study title; publication date; country; study type; outcome type; outcome effect size; sample size; and participant characteristics.

### Quality assessment

Risk of bias was assessed by two independent researchers (RD; EI) using the relevant Joanna Briggs Institute (JBI) tools for cross-sectional [23] and case-control [24] studies. Broadly, the JBI tools are non-scoring appraisal tools for assessing the methodological quality of a study and to determine the extent to which a study has addressed the possibility of bias in its design, conduct and analysis. Any discrepancies over the final risk of bias verdict were solved by consensus, with involvement of a third review author (MT) where necessary.

### Statistical analysis

Two random-effects meta-analyses were conducted using the DerSimonian and Laird method, with studies weighted according the inverse variance, using Comprehensive Meta-Analysis [25]. The first meta-analysis pooled ORs of STDR and NSTDR in populations with versus without vitamin D deficiency (defined as 25(OH)D levels of <20 ng/mL). The second meta-analysis calculated the standard mean difference (SMD) of 25(OH)D levels in participants with either STDR or NSTDR against participants with diabetes but no evidence of DR. Heterogeneity between studies was assessed using the I^2^ statistic, with 0–50% being classified as low, 50–75% as moderate, and > 75% classified as high heterogeneity [26]. Publication bias was assessed with a visual inspection of funnel plots. Furthermore, sensitivity analyses were conducted to assess the (a) robustness of analyses, and (b) potential sources of heterogeneity, through the one study removed method.

### Certainty of evidence

To ascertain the certainty of the evidence, the Grading of Recommendations, Assessment, Development and Evaluations [27, 28] (GRADE) framework was used (see Supplementary Table 3 for full information).

## Results

**Fig. 1:**
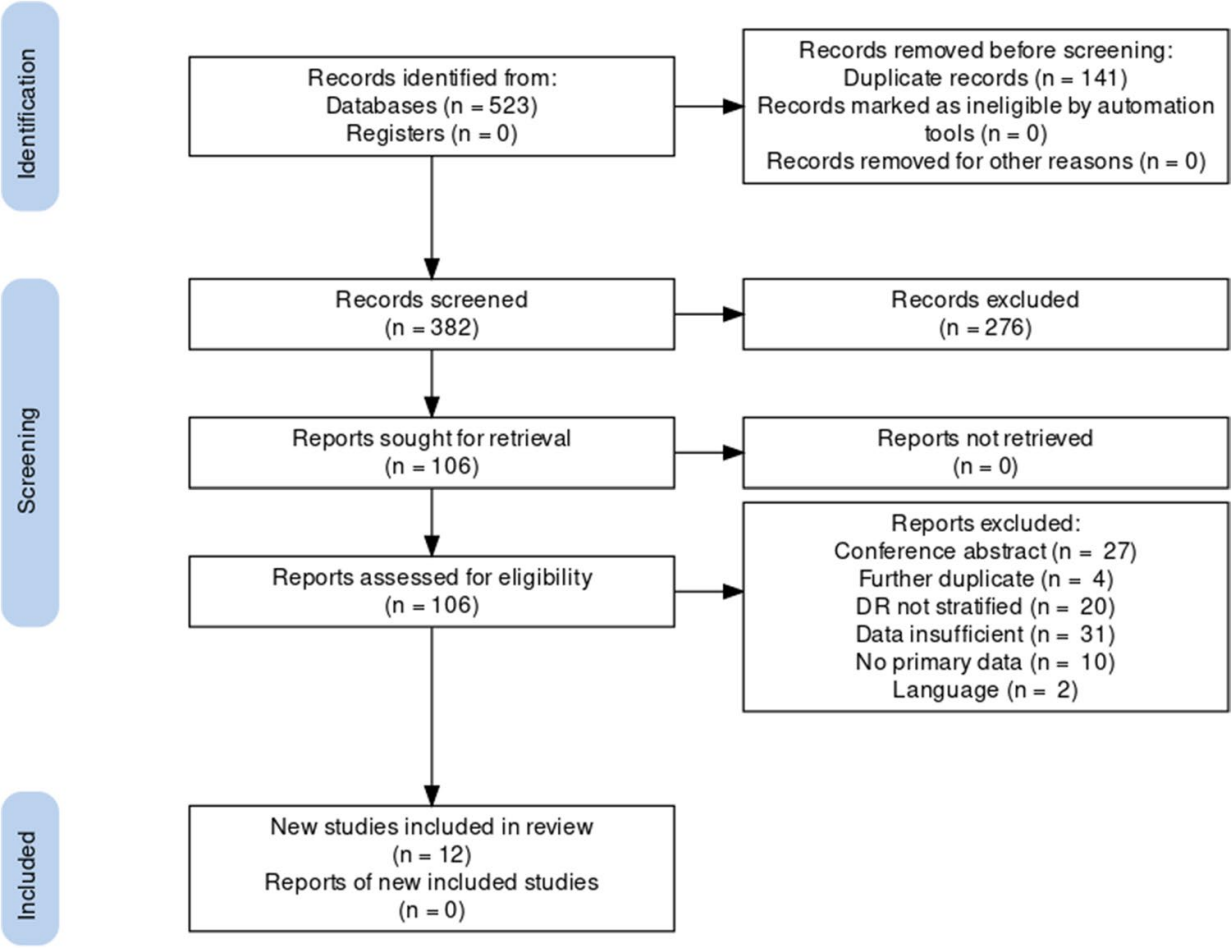
PRISMA flowchart of included studies

The initial search yielded 523 articles, of which 141 were automatically removed, leaving 382 articles for title and abstract screening. Of these 382 articles, 106 were selected for full text screening. Following full text screening, 12 studies (*n* = 9057) were included in the meta-analysis [20, 29–39], with descriptive characteristics in Table 1. Studies were excluded for several reasons, including being conference abstracts, having insufficient data, and not stratifying the type of DR. A list of excluded studies with justifications can be found in Supplementary Table 1. The full PRISMA flowchart can be found in Fig. 1. Most included studies (*n* = 11) were cross-sectional, with one case-control study. All studies were deemed to have a low risk of bias (full JBI scoring in Supplementary Table 2).

**Table 1:** Descriptive characteristics of included studies

Authors	Study design	Country	Mean age (SD)	Percentage female	Total n	Type of DM
Aksoy et al.	Cross sectional	Turkey	NR	NR	66	Type 2
Alam et al.	Cross sectional	UK	No DR = 59.8 (13.8) BDR = 58.8 (13.3) Pre PDR = 60.8 (10.9) PDR = 55.1 (13.6)	49.5	657	Mixed
Alcubierre et al.	Case-control	Spain	NR	NR	427	NR
Almoosa et al.	Cross sectional	Bahrain	NR	NR	662	Type 2
Ashinne et al.	Cross sectional	India	55.3 (10.2)	38.2	3054	Type 2
Bonakdaran and Nasser	Cross sectional	Iran	54.8 (9.4)	69.8	235	Type 2
He et al.	Cross sectional	China	59.03 (11.67)	49.14	1520	Type 2
Long et al.	Cross sectional	USA	61.24 (SE = 0.46)	52.8	1293	NR
Kim et al.	Cross-sectional	South Korea	No DME = 62.2(10.5) DME = 57.7(10.1)	No DME = 60; DME = 37	65	NR
Nadri et al.	Cross sectional	India	No DR = 53.24(1.2) NPDR = 53.72(1.4) PDR = 53.61(1.7)	No DR = 59 NPDR = 27 PDR = 41	66	Type 2
Payne et al.	Cross sectional	USA	No DR = 62.4(11.3) NPDR = 68.3 (10.0) PDR = 59.8 (12.0)	No DR = 49 NPDR = 47 PDR = 50	123	NR
Zhou et al.	Cross sectional	China	No DR = 57.65 (11.49) DR = 58.10 (10.83)	No DR = 41.2%; DR = 47.6%	889	NR

*DM* diabetes mellitus, *SD* standard deviation, *NR* not reported, *DR* diabetic retinopathy, *BDR* background diabetic retinopathy, *PDR* proliferative diabetic retinopathy, *NPDR* non-proliferative diabetic retinopathy, *DME* diabetic macular edema

### Vitamin D deficiency

**Fig. 2:**
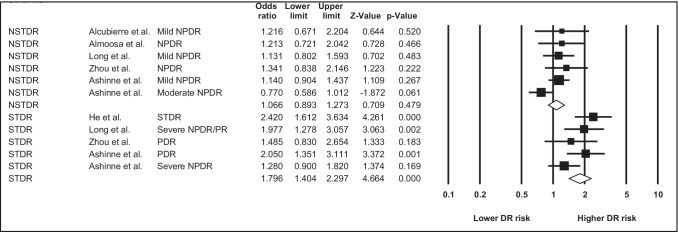
Forest plot showing associations between vitamin D deficiency and the risk of non-sight threatening versus sight threatening diabetic retinopathy

When dichotomising vitamin D status into deficiency (25(OH)D levels of <20 ng/mL) versus non-deficiency, 6 studies were included in the meta-analysis. The meta-analysis showed that NSTDR was not significantly associated with vitamin D deficiency (OR = 1.10 95%CI 0.90–1.27; *p* = 0.48; I^2^ = 30.21), whereas STDR was significant associated with vitamin D deficiency (OR = 1.80 95%CI 1.40–2.30; *p* = <0.001; I^2^ = 39.39), see Table 2 and Fig. 2 for more details. When assessing funnel plots, no publication bias was observed in either sub-group (see Supplemental Figs. 1 and 2). The significance and magnitude of results were not affected by the removal of any one study. This level of evidence was rated as ‘low’ according to the GRADE criteria, predominately because of the included studies were observational in design, despite low heterogeneity, and robustness of results when one study removed (see Supplemental Table 4).

**Table 2:** Meta-analysis showing the risk of diabetic retinopathy in vitamin D deficiency versus no deficiency

Type of DR	N studies	OR (95% CI)	p value	Heterogeneity
NSTDR	5 (6 outcomes)	1.066 (0.893–1.273)	0.479	30.206
STDR	4 (5 outcomes)	1.796 (1.404–2.297)	<0.001	39.392

*DR* diabetic retinopathy, *NSTDR* non-sight threatening diabetic retinopathy, *STDR* sight threatening diabetic retinopathy, *OR* odds ratio, *CI* confidence interval

### 25(OH)D levels

**Fig. 3:**
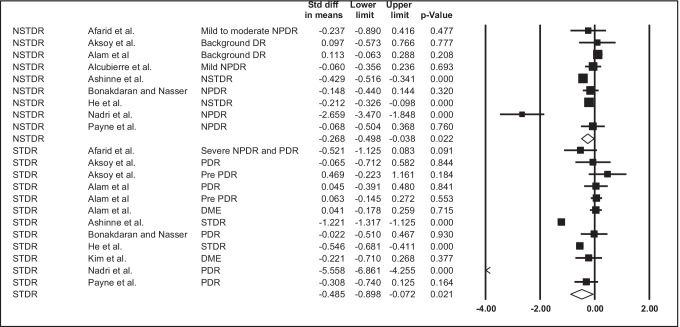
Forest plot showing standard differences in means in 25(OH)D levels between people with versus without diabetic retinopathy

When considering vitamin D as a continuous variable, 9 studies (yielding 21 outcomes) were included in the meta-analysis. Both NSTDR and SRDR were significantly associated with 25(OH)D levels (NSTDR SMD = -0.27 95%CI -0.50; −0.04; *p* = 0.02; I^2^ = 88.51; STDR SMD = −0.49 95%CI -0.90;-0.07; *p* = 0.02; I^2^ = 96.42), see Table 3 and Fig. 3. There was some evidence of publication bias when observing the funnel plots (see Supplementary Figs. 3 and 4). In the NSTDR sub-group, the removal of either Ashinne et al. [33] or Nadri et al. [37] changed the significance of results. Furthermore, the removal of Ashinne et al. [33] reduced heterogeneity in the NSTDR group from 88.52 to 69.14. In the STDR group, the removal of Nadri et al. [37] changed the significance of results. Because of the high heterogeneity, possible publication bias, lack of robust results as shown in the sensitivity analyses (see Supplemental Table 5), and observational nature of studies, the credibility of this evidence has been rated as ‘low’, according to the GRADE criteria.

**Table 3:** Meta-analysis showing standard mean differences between 25(OH)D levels in people with versus without diabetic retinopathy

Type of DR	N studies	SMD (95% CI)	*p* value	Heterogeneity
NSTDR	9 (9 outcomes)	−0.268 (−0.498; −0.038)	0.022	88.514
STDR	9 (12 outcomes)	−0.485 (−0.898; −0..072)	0.021	96.417

*DR* diabetic retinopathy, *NSTDR* non-sight threatening diabetic retinopathy, *STDR* sight threatening diabetic retinopathy, *SMD* standard mean difference, *CI* confidence interval

## Discussion

This systematic review, which included 12 studies and 9057 participants, reported associations between vitamin D status and NSTDR/STDR. The results indicate that vitamin D deficiency (25(OH)D < 20 ng/mL) significantly increases the odds of STDR by a magnitude of 1.8 but is not associated with NSTDR risk. Earlier reports show lower odds ratios than those found in this study, however these examined PDR only and yielded ORs of 1.69 and 1.32 respectively [20, 21]. Our results show high level evidence that a significantly higher OR is associated for vitamin D deficiency when DME is also included, backed by lower heterogeneity than previous reviews.

The finding that NSTDR was not associated with vitamin D deficiency does not agree with Zhang et al. [21], who found a significant association between vitamin D deficiency and NPDR. The results of this study, however, agree with much of the literature that suggests an inverse relationship with vitamin D levels and severity of DR [21, 29]. A possible reason for the lack of agreement with Zhang et al. may be because this study included severe NPDR as a form of STDR, which was categorised in this review as NPDR, in line with previous research.

Possible mechanisms to explain the association between vitamin D deficiency and STDR have not been extensively examined, however there are several possible mechanisms. For example, several studies have found associations between vitamin D receptor genes and DR, with both the *BsmI* polymorphism B allele and the F allele of the *FokI* vitamin D receptor gene being linked to DR prevalence [21, 40, 41]. Moreover, vitamin D supplementation has been suggestively linked to improved glycaemic control [42, 43]. Further study is warranted to explore the role of the vitamin D receptor in the progression of DR.

Whilst showing important conclusions, the limitations of this review should be considered. Firstly, the methodology of included studies precludes the establishment of causal relationships – further longitudinal and interventional studies are warranted to determine causality between vitamin D deficiency and the progression of DR. Secondly, there was high heterogeneity that could not be fully explored, mainly due to a relatively small number of studies. Several studies were excluded from the analysis because they had not categorised DR into any form of sub-group. It is recommended that future research stratifies DR in sub-groups so that further reviews can include a larger amount of data. Furthermore, due to the small number of studies, we did not consider the geographical location and time of the year in studies, which have been shown to be a factor in vitamin D synthesis [44, 45]. Lastly, the lack of adjusting for cofounding variables (such as age, duration of diabetes and hypertension etc) should be taken into consideration (although the two included studies that did adjust for these variables in multivariable analyses yielded similar results). It is recommended that multivariable analysis be used in primary studies wherever possible.

Despite these limitations, this study provides robust evidence of a significant relationship between vitamin D deficiency and STDR. Because vitamin D deficiency is associated with other unfavourable outcomes (such as mortality), and there is evidence that vitamin D status is associated with sight threatening stages of retinopathy – it is recommended that vitamin D levels be regularly screened in people with diabetes, and that vitamin D be supplemented when needed, so that deficiency is prevented.

## Conclusion

Vitamin D deficiency is significantly associated with STDR, but not with NSTDR. Given the well-reported associations between vitamin D deficiency and other unfavourable outcomes, it is important that vitamin D deficiency is managed appropriately and in a timely manner to reduce the risk of blindness in people with diabetes.

## Supplementary Information

ESM 1 (DOCX 66 kb)
