# Supplementary material for: Associations between vitamin D status and sight threatening and non-sight threatening diabetic retinopathy: a systematic review and meta-analysis
Source: J Diabetes Metab Disord. 2022 May 26;21(1):1177–84. doi: 10.1007/s40200-022-01059-3 (PMC9167360; doi:10.1007/s40200-022-01059-3)
Supplement: Supplementary file 1 — (DOCX 66 kb) [file 40200_2022_1059_MOESM1_ESM.docx]

**Supplementary Tables and Figures**

 **Supplementary Figure 1: Funnel plot showing standard error and the log of the odds ratio of the non-sight threatening diabetic retinopathy meta-analysis sub-group.**

 **Supplementary Figure 2: Funnel plot showing standard error and the log of the odds ratio of the sight threatening diabetic retinopathy meta-analysis sub-group**

 **Supplementary Figure 3: Funnel plot showing standard error and the log of the standard difference in means of the non-sight threatening diabetic retinopathy meta-analysis sub-group**

 **Supplementary Figure 4: Funnel plot showing standard error and the log of the standard difference in means of the sight threatening diabetic retinopathy meta-analysis sub-group**

**Supplementary Table 1: Excluded studies with rationale**

| **Author(s)** | **Title** | **Reason for exclusion** |
| --- | --- | --- |
| Abdella et al. | Does Vitamin D deficiency play a role in peripheral neuropathy in Type 2 diabetes? | No diabetic retinopathy data |
| Ahmadieh et al. | Hypovitaminosis D in patients with type 2 diabetes mellitus: A relation to disease control and complications | DR type not stratified |
| Ahmadieh et al. | Hypovitaminosis d in patients with type 2 diabetes mellitus: Relationship with microvascular complications | Conference abstract |
| Ahmed et al. | Relationship between total vitamin d metabolites and complications in patients with type 2 diabetes | Data presented as median - unable to pool |
| Ahmed et al. | Vitamin D3 metabolite ratio as an indicator of vitamin D status and its association with diabetes complications | Data presented as median - unable to pool |
| Ahmed et al. | Association of vitamin D2 and D3 with type 2 diabetes complications | DR type not stratified |
| Alcubierre et al. | Vitamin D deficiency is associated with poorer satisfaction with diabetes-related treatment and quality of life in patients with type 2 diabetes: a cross-sectional study. | Did not stratify DR |
| Alele et al. | Relationship between vitamin D status and incidence of vascular events in the veterans affairs diabetes trial | Retinopathy data not stratified by Vit D |
| Amjad et al. | Vitamin D deficiency is not associated with diabetic retinopathy DUPLICATE OF LINE 13 | Duplicate |
| Atkin et al. | Association of vitamin d metabolites with type 2 diabetes complications in Qatar | Conference abstract |
| Bajaj et al. | Vitamin D levels and microvascular complications in type 2 diabetes | Did not stratify DR |
| Bener et al. | The Impact of Vitamin D Deficiency on Retinopathy and Hearing Loss among Type 2 Diabetic Patients | Control group insufficient? |
| Bowie et al. | Vitamin D and diabetic retinopathy | Conference abstract |
| Brix et al. | Vitamin D is not associated with diabetic retinopathy in patients with type 1 diabetes and type 2 diabetes | Conference abstract |
| Brunetti et al. | Association between vitamin D deficiency and diabetic retinopathy in the third national health and nutrition examination survey | Conference abstract |
| Butler et al. | Association of vitamin D3 and its metabolites in patients with and without type 2 diabetes and their relationship to diabetes complications | Data presented as median - unable to pool |
| Castillo-Oti et al. | Prevalence and risk factors associated with diabetic retinopathy in Santander. Northern Spain | No diabetic retinopathy data |
| Castillo-Oti et al. | ﻿Vitamin D Deficiency Is Significantly Associated with Retinopathy in Type 2 Diabetes Mellitus: A Case-Control Study | DR type not stratified |
| Castro et al. | The Role of Vitamin D in the Health of Hispanic Adults With Diabetes. | No primary data |
| Cusumano et al. | HLA genotype and EV infection at diagnosis of type 1 diabetes predicts vascular complications in type 1 diabetes | Conference abstract |
| Cusumano et al. | Vitamin D deficiency is not associated with changes in retinal geometric parameters in young people with type 1 diabetes | DR type not stratified |
| Cyganek et al. | Clinical risk factors and the role of VDR gene polymorphisms in diabetic retinopathy in Polish type 2 diabetes patients | No vitamin D data |
| Dahan et al. | HAPTOGLOBIN POLYMORPHISM AS AN INDEPENDENT PREDICTOR OF DIABETIC NEPHROPATHY AND RETINOPATHY | Article in Hebrew |
| Darraj et al. | Vitamin D deficiency and glycemic control among patients with type 2 diabetes mellitus in Jazan City, Saudi Arabia | DR type not stratified |
| De Almeida et al. | The association between Vitamin D receptor gene polymorphisms (Taqi and Foki) and micro/macrovascular complications in postmenopausal women with type 2 diabetes | No diabetic retinopathy data |
| Do Nascimento et al. | Effect of high Vitamin D doses on diabetic retinopathy in patients with type 1 diabetes mellitus | Conference abstract |
| Garcia et al. | Hypovitaminosis D and microvascular complications in diabetic patients | Conference abstract |
| Gebhart | Low vitamin D levels may be associated with diabetes; supplementation trials needed. | Editorial |
| Gungor et al. | Retinal nerve fiber layer thickness in early-stage diabetic retinopathy with vitamin D deficiency | DR type not stratified |
| Herrmann et al. | Serum 25-Hydroxyvitamin D: A predictor of macrovascular and microvascular complications in patients with type 2 diabetes | DR type not stratified |
| Horikawa et al. | Higher dietary intake of vitamin d is associated with lower incidence of diabetic nephropathy in Japanese patients with type 2 diabetes | Conference abstract |
| Hussain et al. | An epidemic of vitamin D deficiency in the central Manchester population: Relationship to diabetes complications and ethnicity | Conference abstract |
| Hyeon hong et al. | Association of serum 25-hydroxyvitamin d with low level of HDL in patients with type 2 diabetes mellitus | Conference abstract |
| Chi | Patients with diabetes complications were treated by Vitamin D | Conference abstract |
| IRCT20200407046978N1 | Evaluation of vitamin D effect on diabetic macular edema | Trial protocol |
| Jee et al. | Serum 25-hydroxyvitamin D levels and dry eye syndrome: Differential effects of Vitamin D on ocular diseases | Data insufficient |
| Jiao et al. | Association of FokI, TaqI, BsmI and ApaI polymorphisms with diabetic retinopathy: a pooled analysis of case-control studies | No primary data |
| Joergensen et al. | Vitamin D levels, microvascular complications, and mortality in type 1 diabetes | Data insufficient |
| Josef et al. | Concentration of NK cells after beta-glucan and vitamin D supplementation in patients with diabetic retinopathy | No diabetic retinopathy data |
| Jung et al. | Relationship between vitamin D status and vascular complications in patients with type 2 diabetes mellitus | DR type not stratified |
| Kaur et al. | Vitamin D deficiency is associated with retinopathy in children and adolescents with type 1 diabetes | DR type not stratified |
| Khan et al. | Association of vitamin D with retinopathy in patients with type 2 diabetes mellitus | DR type not stratified |
| Khan et al. | Vitamin D deficiency in patients with diabetic retinopathy | DR type not stratified |
| Kim et al. | Inverse association between high blood 25-hydroxyvitamin D levels and diabetic retinopathy in a representative Korean population | Did not stratify DR |
| Kim et al. | Serum and aqueous humor vitamin D levels in patients with diabetic macular edema | not DR |
| Kirac et al. | VDBP, VDR mutations and other factors related with vitamin D metabolism may be associated with type 1 diabetes mellitus | Data insufficient |
| Koch et al. | Autophagy in diabetic nephropathy: a review | No primary data |
| Kucukler et al. | Relationship between 25 (OH) vitamin D and microvascular complications of type 2 diabetes mellitus | Conference abstract |
| Li et al. | The relationship between serum vitamin D and diabetic retinopathy in Chinese community type 2 diabetic patients | Conference abstract |
| Lopes et al. | Association between serum Vitamin D and diabetic retinopathy in Portuguese patients with type 1 diabetes | Not stratified by DR type |
| Luo et al. | The association between vitamin D deficiency and diabetic retinopathy in type 2 diabetes: A meta-analysis of observational studies | No primary data |
| Maia et al. | The association between vitamin D receptor gene polymorphisms (Taqi and Foki), Type 2 diabetes, and micro-/macrovascular complications in postmenopausal women | Duplicate |
| Maamar et al. | ﻿Trabecular bone score and 25-hydroxyvitamin D levels in microvascular complications of type 2 diabetes mellitus | Not stratified by DR type |
| Millen et al. | Vitamin D status and diabetic retinopathy in a biracial cohort | Conference abstract |
| Oh et al. | The association between Bsm1/Apa1 polymorphisms in the vitamin D receptor gene and complications of type 2 diabetes in the Korean population | Conference abstract |
| Omidian et al. | Effects of vitamin D supplementation on circulatory YKL-40 and MCP-1 biomarkers associated with vascular diabetic complications: A randomized, placebo-controlled, double-blind clinical trial | No diabetic retinopathy data |
| Park | Serum and aqueous humor vitamin D levels in diabetic macular edema patients | Conference abstract |
| Patrick et al. | Vitamin d and retinopathy in adults with diabetes mellitus. | Data reported in medians |
| Poon et al. | Vitamin D deficiency is not associated with retinal vascular calibre | Duplicate |
| Poon et al. | Are environmental and genetic factors at type 1 diabetes diagnosis associated with the development of microvascular complications? | Conference abstract |
| Reddy et al. | Plasma vitamin D status in patients with type 2 diabetes with and without retinopathy | DR type not stratified |
| Reheem et al. | Serum vitamin D and parathormone (PTH) concentrations as predictors of the development and severity of diabetic retinopathy | wrong type of VD measure used |
| Richter et al. | Effects of beta-glucan and Vitamin D Supplementation on Inflammatory Parameters in Patients with Diabetic Retinopathy | No diabetic retinopathy data |
| Rodrigues et al. | Evaluation of 25-hydroxivitamin D levels and Vitamin D receptor polymorphisms in type 2 diabetes mellitus patients | Conference abstract |
| Rui et al. | Vitamin D deficiency increases the risk of retinopathy in Chinese patients with type 2 diabetes DUPLICATE OF 88 | Duplicate |
| Salvia et al. | Vitamins and functional nutrients for extremely low birth weight infants | Conference abstract |
| Saxena et al. | Low serum vitamin D levels correlate with disorganization of retinal inner layers, ellipsoid zone disruption and retinal pigment epithelium alterations in diabetic retinopathy | Conference abstract |
| Selam et al. | Association between a protein polymorphism in the start codon of the vitamin D receptor gene and severe diabetic retinopathy in C-peptide-negative type 1 diabetes | No vitamin D data |
| Senyigit | The association between 25-hydroxy vitamin D deficiency and diabetic complications in patients with type 2 diabetes mellitus | DR type not stratified |
| Shepelkevich et al. | Vitamin D level in type 1 diabetic patients | Conference abstract |
| Shimo et al. | Vitamin D deficiency is significantly associated with retinopathy in young Japanese type 1 diabetic patients | DR type not stratified |
| Soesanti et al. | High prevalence of vitamin D deficiency among adolescents with type 1 diabetes in Indonesia and its association with diabetic retinopathy and nephropathy | Conference abstract |
| Song et al. | The Impact of Vitamin D Receptor Gene Polymorphisms on the Susceptibility of Diabetic Vascular Complications: A Meta-Analysis | No primary data |
| Sugano et al. | Vitamin D deficiency is related to low eicosapentaenoic acid to arachidonic acid ratio in type 2 diabetes | Conference abstract |
| Tan et al. | Serum 25-hydroxy-vitamin D levels and diabetic retinopathy in a multi-ethnic Asian population | Conference abstract |
| Taverna et al. | Taq I polymorphism of the vitamin D receptor and risk of severe diabetic retinopathy | No diabetic retinopathy data |
| Tecilazich et al. | Role of vitamin D in diabetic retinopathy: Pathophysiological and clinical aspects | No primary data |
| Tougaard et al. | The association between vitamin D level and microvascular complications in persons with type 2 diabetes | Data reported in medians |
| Valle et al. | Protective role of vitamin D against oxidative stress in diabetic retinopathy | No primary data |
| Varun Kumar et al. | Vitamin D levels and prevalence of complications in type 2 diabetes | Conference abstract |
| Wan et al. | Associations between Vitamin D and microvascular complications in middle-aged and elderly diabetic patients | DR type not stratified |
| Wong et al. | A systematic review of the associations between dietary intake and diabetic retinopathy | Conference abstract |
| Wong et al. | Dietary intake and diabetic retinopathy: A systematic review | No primary data |
| Xiao et al. | Association Between Vitamin D Status and Diabetic Complications in Patients With Type 2 Diabetes Mellitus: A Cross-Sectional Study in Hunan China | Not stratified by DR type |
| Yildiz et al. | Effect of serum 25 hydroxy Vitamin D level on macular edema in patients with nonproliferative diabetic retinopathy | DR type not stratified |
| Zavorkova et al. | Effects of glucan and vitamin D supplementation on obesity and lipid metabolism in diabetic retinopathy | No diabetic retinopathy data |
| Zhang et al. | Relationship between vitamin D deficiency and diabetic retinopathy: a meta-analysis | No primary data |
| Zhang et al. | The Association between VDR Gene Polymorphisms and Diabetic Retinopathy Susceptibility: A Systematic Review and Meta-Analysis | No primary data |
| Zhao et al. | Relationship of serum vitamin D levels with diabetic microvascular complications in patients with type 2 diabetes mellitus | DR type not stratified |
| Zhong et al. | Effects of vitamin D receptor gene polymorphism and clinical characteristics on risk of diabetic retinopathy in Han Chinese type 2 diabetes patients | No vitamin D data |
| Zhu et al. | Relationship between serum vitamin D levels and diabetes retinopathy in type 2 diabetes mellitus patients | Article in Chinese |
| Zoppini et al. | Lower levels of 25-hydroxyvitamin D3 are associated with a higher prevalence of microvascular complications in patients with type 2 diabetes | Data insufficient |

**Supplementary Table 2: JBI scoring**

| **Authors** | **JBI critical appraisal questions** | | | | | | | | | |
| --- | --- | --- | --- | --- | --- | --- | --- | --- | --- | --- |
|  | **1** | **2** | **3** | **4** | **5** | **6** | **7** | **8** | **9** | **10** |
| Aksoy et al. | Y | Y | Y | Y | Y | N | Y | Y | NA | NA |
| Alam et al. | Y | Y | Y | Y | Y | Y | Y | Y | NA | NA |
| Alcubierre et al.* | Y | Y | Y | Y | Y | N | N | Y | Y | Y |
| Almoosa et al. | Y | N | Y | Y | N | N | Y | N | NA | NA |
| Ashinne et al. | Y | Y | Y | Y | Y | Y | Y | Y | NA | NA |
| Bonakdaran and Shoeibi | Y | Y | Y | Y | N | N | Y | Y | NA | NA |
| He et al. | Y | Y | Y | Y | Y | Y | Y | Y | NA | NA |
| Long et al. | Y | Y | Y | N | Y | Y | Y | Y | NA | NA |
| Nadri et al. | Y | Y | Y | Y | Y | N | Y | Y | NA | NA |
| Payne et al. | Y | Y | Y | Y | Y | Y | Y | Y | NA | NA |
| Zhou et al. | Y | Y | Y | Y | Y | Y | Y | Y | NA | NA |
| Kim et al. | Y | Y | Y | Y | Y | Y | Y | Y | NA | NA |

*Note that Alcubierre et al. was a case-control study and used the JBI case-control critical appraisal tool.

**Supplementary Table 3: GRADE Scoring criteria**

| **Study design** | **Initial quality of a body of evidence** | **Lower if** | **Higher if** | **Quality of a body of evidence** |
| --- | --- | --- | --- | --- |
| Randomized trials | High 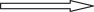 | Risk of Bias  −1 Serious  −2 Very serious  Inconsistency  −1 Serious  −2 Very serious  Indirectness  −1 Serious  −2 Very serious  Imprecision  −1 Serious  −2 Very serious  Publication bias  −1 Likely  −2 Very likely | Large effect  +1 Large  +2 Very large  Dose response  +1 Evidence of a gradient  All plausible residual confounding  +1 Would reduce a demonstrated effect  +1 Would suggest a spurious effect if no effect was observed | High (four plus: ⊕⊕⊕⊕) |
|  |  |  |  | Moderate (three plus: ⊕⊕⊕○) |
| Observational studies | Low 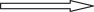 |  |  | Low (two plus: ⊕⊕○○) |
|  |  |  |  | Very low (one plus: ⊕○○○) |

**Supplementary Table 4: Sensitivity analysis for vitamin D deficiency**

|  | Author of removed study | OR if study removed | Lower 95% CI | Upper 95% CI |
| --- | --- | --- | --- | --- |
| STDR | He et al. | 1.649 | 1.293 | 2.104 |
|  | Long et al. | 1.757 | 1.287 | 2.398 |
|  | Zhou et al. | 1.855 | 1.391 | 2.474 |
|  | Ashinne et al. | 1.738 | 1.277 | 2.364 |
|  | Ashinne et al. | 2.035 | 1.628 | 2.545 |
| NSTDR OR | Alcubierre et al. | 1.059 | 0.867 | 1.294 |
|  | Almoosa et al. | 1.056 | 0.862 | 1.293 |
|  | Long et al. | 1.062 | 0.851 | 1.326 |
|  | Zhou et al. | 1.036 | 0.855 | 1.255 |
|  | Ashinne et al. | 1.053 | 0.833 | 1.331 |
|  | Ashinne et al. | 1.173 | 0.997 | 1.378 |

STDR=sight threatening diabetic retinopathy; NSTDR=non-sight threatening diabetic retinopathy; OR- odds ratio

**Supplementary Table 5: Sensitivity analysis for 25(OH)D levels**

|  | Author of removed study | SMD if study removed | Lower 95% CI | Upper 95% CI |
| --- | --- | --- | --- | --- |
| STDR | Afarid et al. | -0.484 | -0.919 | -0.049 |
|  | Aksoy et al. | -0.521 | -0.953 | -0.089 |
|  | Aksoy et al. | -0.561 | -0.987 | -0.135 |
|  | Alam et al | -0.536 | -0.969 | -0.103 |
|  | Alam et al | -0.539 | -0.966 | -0.111 |
|  | Alam et al. | -0.538 | -0.969 | -0.107 |
|  | Ashinne et al. | -0.351 | -0.694 | -0.008 |
|  | Bonakdaran and Nasser | -0.529 | -0.962 | -0.095 |
|  | He et al. | -0.517 | -1.040 | 0.005 |
|  | Kim et al. | -0.512 | -0.948 | -0.075 |
|  | Nadri et al. | -0.226 | -0.615 | 0.162 |
|  | Payne et al. | -0.505 | -0.944 | -0.066 |
| NSTDR | Afarid et al. | -0.272 | -0.514 | -0.030 |
|  | Aksoy et al. | -0.296 | -0.535 | -0.056 |
|  | Alam et al | -0.326 | -0.561 | -0.091 |
|  | Alcubierre et al. | -0.302 | -0.554 | -0.049 |
|  | Ashinne et al. | -0.256 | -0.533 | 0.021 |
|  | Bonakdaran and Nasser | -0.291 | -0.546 | -0.036 |
|  | He et al. | -0.311 | -0.622 | 0.000 |
|  | Nadri et al. | -0.143 | -0.322 | 0.036 |
|  | Payne et al. | -0.293 | -0.540 | -0.046 |

STDR=sight threatening diabetic retinopathy; NSTDR=non-sight threatening diabetic retinopathy’ SMD = standard mean difference
